# Using principal component analysis to capture individual differences within a unified neuropsychological model of chronic post-stroke aphasia: Revealing the unique neural correlates of speech fluency, phonology and semantics

**DOI:** 10.1016/j.cortex.2016.04.016

**Published:** 2017-01

**Authors:** Ajay D. Halai, Anna M. Woollams, Matthew A. Lambon Ralph

**Affiliations:** Neuroscience and Aphasia Research Unit, University of Manchester, UK

**Keywords:** Individual differences, Principal component analysis, Post-stroke aphasia, Symptom–lesion mapping

## Abstract

Individual differences in the performance profiles of neuropsychologically-impaired patients are pervasive yet there is still no resolution on the best way to model and account for the variation in their behavioural impairments and the associated neural correlates. To date, researchers have generally taken one of three different approaches: a single-case study methodology in which each case is considered separately; a case-series design in which all individual patients from a small coherent group are examined and directly compared; or, group studies, in which a sample of cases are investigated as one group with the assumption that they are drawn from a homogenous category and that performance differences are of no interest. In recent research, we have developed a complementary alternative through the use of principal component analysis (PCA) of individual data from large patient cohorts. This data-driven approach not only generates a single unified model for the group as a whole (expressed in terms of the emergent principal components) but is also able to capture the individual differences between patients (in terms of their relative positions along the principal behavioural axes). We demonstrate the use of this approach by considering speech fluency, phonology and semantics in aphasia diagnosis and classification, as well as their unique neural correlates. PCA of the behavioural data from 31 patients with chronic post-stroke aphasia resulted in four statistically-independent behavioural components reflecting phonological, semantic, executive–cognitive and fluency abilities. Even after accounting for lesion volume, entering the four behavioural components simultaneously into a voxel-based correlational methodology (VBCM) analysis revealed that speech fluency (speech quanta) was uniquely correlated with left motor cortex and underlying white matter (including the anterior section of the arcuate fasciculus and the frontal aslant tract), phonological skills with regions in the superior temporal gyrus and pars opercularis, and semantics with the anterior temporal stem.

## 1 Introduction

For both clinical practice and basic research, it is important to understand the patterns and neural bases of impaired performance observed in both individual and groups of neuropsychological patients. Since the beginning of behavioural neurology and neuropsychology, contrastive impairments across patients have been used to make important inferences about the underlying cognitive computations and the neural structures that support them. In addition, stable models of variable patient performance are critical in accurate neurological differential diagnosis, clinical management and treatment.

The generation of stable, reliable models of normal and impaired function rely on the ability to understand the nature and sources of individual differences across patients. This has always been a key challenge for the field (Shallice, 1988), leading to numerous debates and discussions (Caramazza and McCloskey, 1988, Lambon Ralph et al., 2011, Schwartz and Dell, 2011, Shallice, 1988), and arguably still is. The kernel of the problem relates to the fact that there are multiple sources that underlie variable neuropsychological results. Previous formal considerations of the issue have set out a series of potential factors (Lambon Ralph et al., 2002, Shallice, 1988) but, for brevity, we will note three types here: (A) the type of difference that neuropsychological dissociations and differential diagnosis are based on, namely stable performance differences that arise when a task is supported by one or more computations underpinned by neuroanatomically-separated regions; (B) differences that relate, linearly or nonlinearly, to the severity of damage to the underlying neurocomputational component(s) – which can provide critical information about the nature and characteristics of the core neurocognitive computations, as well as the degree of recovery/deterioration in a patient's disease or disorder; and (C) sources of individual differences of no interest including random variations, measurement noise, and so forth.

Over the years, researchers have adopted different approaches to this issue. The most classical and common approach (also utilised by experimental psychology and functional neuroimaging) is to recruit a coherent sample of patients and, through the power of central tendency, average the behavioural results or lesion distributions in order to remove noise and other individual variations of no interest. As noted previously (Shallice, 1988), this method relies on having or defining a ‘coherent’ group and, at worst, can suffer from two types of statistical error: (a) failing to uncover meaningful differences within the sample of patients which is lost through the averaging process; and (b) failing to detect or understand how systematic variation in patient/lesion severity changes task performance (which otherwise is falsely considered to reflect a behavioural dissociation). An alternative approach, single-case study, possesses the opposite set of advantages and disadvantages. Of course, whilst single-case study avoids averaging away important behavioural dissociations, it means that it is hard/impossible to place each patient within a broader context or to generate a coherent, overarching cognitive and neuroanatomical model of the disorder. From a basic science perspective, this approach could lead to the logical absurdity of there being as many theories as there are patients; whilst, from a clinical perspective, it reduces our ability to generalise clinical knowledge from one patient to another – ultimately making it impossible to successfully diagnose, manage and treat the patient's impairments. The single-case study makes it impossible to map the relationship between severity of damage and level of performance (which, like any mathematical function, requires more than one datum). Over the past decade or so, multiple researchers have adopted a hybrid of these two approaches in the form of case-series studies (Hodges et al., 1992, Lambon Ralph et al., 2001, Schwartz et al., 2006, Woollams et al., 2007). These investigations recruit a sample of patients whom are all studied in detail at the individual level (i.e., often to the same precision as single-case studies), which can be used: to assess the consistency of performance, i.e., the coherence, in the sampled cases (Lambon Ralph et al., 2001, Schwartz et al., 2006, Woollams et al., 2007); to highlight patterns/mechanisms/lesion correlates which generalise reliably across cases; to identify meaningful, consistent patterns of individual differences (rather than random noise: Woollams et al., 2007); as well as to relate impairment severity to task performance (leading to mathematical/computational models of the severity-based functions: Lambon Ralph et al., 2001, Schwartz et al., 2006, Woollams et al., 2007). Furthermore, ‘comparative’ case-series can be used to strengthen important behavioural dissociations by demonstrating that, despite variations in severity, (i) the two case-series are systematically different in the expected way, yet (ii) are internally coherent (Jefferies and Lambon Ralph, 2006, Lambon Ralph et al., 2007).

Whilst the case-series methodology clearly has a number of strengths which benefit basic science and clinical practice, like group studies, the approach still relies heavily both on the ability to recruit reasonably coherent patient groups and also to know, *a priori*, what the relevant groupings of patient are. Indeed, we note here that one of the most common uses and development platforms of the case-series methodology were made through investigations of semantic dementia (Hodges et al., 1992, Lambon Ralph et al., 2001, Woollams et al., 2007), which is a highly consistent group of patients and thus makes this methodology particularly successful and powerful. It is in this context, therefore, that we consider a related approach – the use of principal component analysis (PCA) as a data-driven method which uses the patterns of individual differences in order both to reveal the statistically-reliable distinctions within a patient dataset and also to place individual cases, relative to each other, in the resultant multi-dimensional model (Butler et al., 2014, Lambon Ralph et al., 2002, Lambon Ralph et al., 2003). In effect, the PCA method spans group and individual levels of analysis because the emergent set of principal components provide a coherent, generalisable set of factors or model of the underlying systems whilst, unlike group studies, variations in individual patient performance can be placed systematically along one or more of the principal axes. If a group is relatively homogenous then PCA only generates one principal component along which all patients can be mapped. If, however, there are systematic differences within the cohort, then one or more statistically-independent (orthogonal) factors emerge. In contrast unsystematic sources of variation, such as measurement error and other random fluctuations are left as unexplained variance (or loaded onto many additional components with little explanatory power, i.e., with eigenvalues less than 1). In addition, in more recent work, we have demonstrated that the PCA factors can also be used to explore behaviour–brain correlates in a new fashion; rather than traditional lesion-overlapping methods which rely on coherent group studies (Caramazza and McCloskey, 1988, Shallice, 1988): or correlating raw test scores with brain voxel status (as per lesion–symptom mapping: Bates et al., 2003), we have found that clear results emerge when the behavioural principal components are related to the patients' lesion distributions (Butler et al., 2014, Chase, 2014).

In this study, therefore, we demonstrate and use the PCA approach in order to explore the nature of post-stroke aphasia. Aphasia is an interesting test case/problem for multiple reasons. Whilst it is possible to distinguish between patients with and without acquired language impairments, within the aphasic group there are multiple sources of individual differences. Traditionally, category-based classification schemes have been developed in order to assist with different diagnosis of aphasic subgroups, clinical management and interventions, as well as for basic science studies that have tried to relate aphasic profiles to underlying lesion distributions. Whilst these category-based systems may provide a useful ‘broad brushstroke’ summary for individual patients, there are multiple limitations which follow from the fact that the underlying distribution of patient data reflects continuous variations along multiple dimensions rather than coherent, mutually-exclusive categories. Instead, there are both very fuzzy boundaries between ‘categories’ and considerable variation of profile within each ‘category’. If this conceptualisation of post-stroke aphasia is correct then we might be able to use PCA in order to define the core underlying dimensions and the relative positions of each individual patient along these axes. Secondly, in turn, this multi-dimensional behavioural description can then be compared directly to the variation in the patients' lesion distributions.

Before considering the PCA findings in this study, we first briefly consider some of the key clinical characteristics of chronic post-stroke aphasia. Speech production, phonological and semantic impairments are common following left hemisphere stroke and the nature of these deficits forms the principal behavioural divisions in most aphasia classification systems. Typically, patients are first divided into fluent (relatively effortless speech output) or non-fluent types (effortful, slow or reduced complexity and length) and then additionally by the status of their phonological and semantic skills (Goodglass, 1993, Goodglass and Kaplan, 1983, Kertesz, 1982). Despite their prominence in aphasia diagnostics and importance for understanding intact language function, the unique neural correlates of speech dys-/fluency, semantic and phonological deficits remain unclear. Furthermore, when these have been considered, they have been investigated separately and thus the inter-relationship between them is unclear (e.g., is speech fluency, in part, a reflection of patients' phonological or semantic skills?).

Classically, damage to left frontal areas was associated with poor fluency (Broca, 1861); however, studies using high-resolution Magnetic Resonance Imaging (MRI) techniques have found that left frontal lesions do not always result in Broca's aphasia (Basso et al., 1985, Willmes and Poeck, 1993) and production deficits can occur following lesions outside of the frontal lobe, including white matter tracts and the anterior insula (aINS) (Bates et al., 2003, Blank et al., 2002, Damasio, 1992, Dronkers, 1996, Mohr et al., 1978, Wise et al., 1999). Functional neuroimaging studies have identified a broader neural network during connected-speech production. For example, by comparing propositional with automatic speech (e.g., counting), a positron emission tomography (PET) imaging study found extensive left hemisphere activation including the frontal lobe, pre-supplementary motor area (pre-SMA), angular gyrus (AG), fusiform gyrus (FG), and perisylvian areas (Blank et al., 2002). A large-scale left hemisphere speech production network was also identified in a study that measured fluency, complexity and variety of post-stroke aphasic speech (Borovsky, Saygin, Bates, & Dronkers, 2007). These critical sub-components could not be separated when they were related to the patients' neural damage, which hints at the multi-faceted nature of speech fluency. Specifically, considerable anatomical overlap was found across the measures: the number of speech tokens (T) correlated with aINS, pre-motor cortex (PMC); mean length of utterance (MLU) with aINS, PMC and anterior superior temporal gyrus (aSTG); and type–token ratio (TTR) with AG, posterior middle temporal gyrus (pMTG) and aSTG. This outcome probably reflects the inter-correlations between both the behavioural measures and lesion distributions (middle cerebral artery – MCA lesions tend to sample the same subset of brain regions) (Phan, Donnan, Wright, & Reutens, 2005), and because severity-related variance is shared across individual measurements. In addition, it is likely that fluency itself is a multi-faceted aphasic feature (Basilakos et al., 2014), which may benefit from decomposition into unique elements before the relationship with lesions can be satisfactorily explored. Plus, to identify the neural correlates of the full range of aphasic features, it is critical to consider fluency simultaneously alongside phonological and semantic skills.

To achieve this aim, the current study utilised the PCA methodology (Butler et al., 2014, Chase, 2014) to reveal, for the first time, the unique neural correlates of speech fluency, semantic and phonological abilities in chronic post-stroke aphasia. This extends previous findings by taking into account speech fluency measures as part of the wider behavioural profile (phonological and semantic deficits), which is a key dimension on which aphasia patients are typically classified. Specifically, two approaches for tackling the analytical challenges associated with brain–behaviour mapping in chronic aphasia are to derive statistically-independent behavioural factors/dimension from a rotated PCA, and lesion volume as a covariate for anatomical severity. Previous studies using PCA on neuropsychological data have also shown that it offers various benefits. First, there is additional statistical reliability by combining results from multiple tests (Lambon Ralph et al., 2002, Lambon Ralph et al., 2003). Secondly, by maintaining orthogonal components, it is possible to avoid problems of collinearity when undertaking lesion–symptom correlations. Thirdly, the use of varimax rotation promotes a clear cognitive interpretation of each of the principal components. Finally, when the rotated components are combined with lesion–symptom mapping, it is possible to establish the neural correlates of unique components of post-stroke aphasic behaviour (Butler et al., 2014, Mirman et al., 2015a, Mirman et al., 2015b).

In summary, this study investigated the multi-dimensionality of post-stroke aphasic deficits and fluency classification using this novel PCA-lesion mapping approach (Butler et al., 2014). The investigation was conducted in three stages: (i) confirmation of the basic results from a previous focussed-investigation of post-stroke fluency (Borovsky et al., 2007) – which represents a more “standard” approach to brain–behaviour correlation; (ii) decomposition of speech production measures using rotated PCA and identification of the neural correlates; and then, most importantly, (iii) simultaneous decomposition of a large behavioural dataset that included measures of speech production, phonology, semantics and executive abilities using an omnibus PCA, and subsequent identification of their unique neural correlates.

## 2 Methods

### 2.1 Participants

Thirty one chronic stroke patients (either ischaemic or haemorrhagic) were recruited (the same patients as reported by Butler et al., 2014), who had impairment in producing and/or understanding spoken language and their aphasia was classified using the Boston Diagnostic Aphasia Examination (BDAE). No restrictions were placed according to aphasia type or severity (spanning global to minimal aphasia). All patients were at least 12 months post-stroke at the time of scanning and assessment, were native English speakers with normal or corrected-to-normal hearing and vision (see Table 1 for a summary of demographic details). Our selection criteria excluded participants if they had any contraindications for scanning, were pre-morbidly left handed, had more than one stroke or had any other significant neurological conditions. Informed consent was obtained from all participants prior to participation under approval from the local ethics committee. Structural imaging data from a healthy age and education matched control group (eight female, 11 male) were used to determine the lesion outline in the patients using the Seghier, Ramlackhansingh, Crinion, Leff, and Price (2008) lesion identification toolbox.

### 2.2 Neuropsychological assessments and analysis

In order to assess speech production deficits, participants were asked to undertake a picture description task (‘Cookie theft’ from the Boston Diagnostic Aphasia Examination) (Goodglass & Kaplan, 1983), which was audio recorded. Participants were instructed to “tell me everything you see going on in this picture”. In addition to the fluency measures, we utilised an extensive battery of neuropsychological tests to assess participants' language and cognitive abilities (described in Butler et al., 2014), enabling us to understand how speech production measures relate to the patients' input and output phonological, semantic and general executive abilities. These included subtests from the Psycholinguistic Assessments of Language Processing in Aphasia (PALPA) battery (Kay, Lesser, & Coltheart, 1992): auditory discrimination using non-word (PALPA 1) and word minimal pairs (PALPA 2); and immediate and delayed repetition of non-words (PALPA 8) and words (PALPA 9). Tests from the 64-item Cambridge Semantic Battery (Bozeat, Lambon Ralph, Patterson, Garrard, & Hodges, 2000) were included: spoken and written versions of the word-to-picture matching task; Camel and Cactus Test (CCT – picture); and the picture naming test. To increase the sensitivity to mild naming and semantic deficits we used the Boston Naming Test (BNT) (Kaplan, Goodglass, & Weintraub, 1983) and a written 96-trial synonym judgement test (Jefferies, Patterson, Jones, & Lambon Ralph, 2009). The spoken sentence comprehension task from the Comprehensive Aphasia Test (CAT) (Swinburn, Baker, & Howard, 2005) was used to assess sentential receptive skills. The additional cognitive tests included forward and backward digit span (Wechsler, 1987), the Brixton Spatial Rule Anticipation Task (Burgess & Shallice, 1997), and Raven's Coloured Progressive Matrices (Raven, 1962). All scores were converted into percentage. Assessments were conducted with participants over several testing sessions, with the pace and number determined by the participant.

The ‘Cookie theft’ description was recorded and then transcribed. The coding procedure followed the method used by Borovsky et al. (2007). Each utterance was marked and bound morphemes, repetitions, false starts, retraces, unintelligible material and interruptions were coded separately. Repeated and retraced utterances were excluded from analysis and only correct full utterances were coded. When the boundary of an utterance was unclear, or quite lengthy, transcribers applied the rule from Lee (1974) that only one “and” conjunction per sentence was allowed when the “and” connected two independent clauses. From these transcriptions, we extracted the number of word T and types, TTR, number of morphemes and MLU in morphemes. In order to control for the length of response given by each participant, we also computed words-per-minute (WPM). We considered these measures as indices of speech fluency as they are among the simplest to quantify and mirror those commonly used in other studies and clinical practice. There are a number of additional measures that could be used to elaborate the model of fluency, including syntactical features (i.e., see Thompson et al., 2012). These, however, require a more open-ended speech sample and are relatively time-consuming to code. We believe that between the four measures used here, we are capturing the amount of speech output within a confined context. All scores were converted into percentage, where the max score across participants was used.

### 2.3 Principal component analysis

This analysis was split into two parts. First, only the speech production measures were entered into a PCA with varimax rotation (SPSS 16.0). Factors with an eigenvalue exceeding 1.0 were extracted and then rotated. Following orthogonal rotation, the loadings of each test allowed a clear behavioural interpretation of each factor. Individual participants' scores on each extracted factor were then used as behavioural covariates in the neuroimaging analysis. A second PCA was performed on a larger dataset which combined the background neuropsychological data with speech production measures. This allowed us to determine how the speech production factors related to the three previously-extracted (Butler et al., 2014) factors relating to phonology, semantic and executive abilities.

### 2.4 Acquisition of neuroimaging data

High-resolution structural T1-weighted MRI scans were acquired on a 3.0 T Philips Achieva scanner (Philips Healthcare, Best, The Netherlands) using an eight-element SENSE head coil. A T1-weighted inversion recovery sequence with 3D acquisition was employed, with the following parameters: TR (repetition time) = 9.0 msec, TE (echo time) = 3.93 msec, flip angle = 8°, 150 contiguous slices, slice thickness = 1 mm, acquired voxel size 1.0 × 1.0 × 1.0 mm^3^, matrix size 256 × 256, field of view (FOV) = 256 mm, TI (inversion time) = 1150 msec, SENSE acceleration factor 2.5, total scan acquisition time = 575 sec.

### 2.5 Analysis of neuroimaging data

**Fig. 1:**
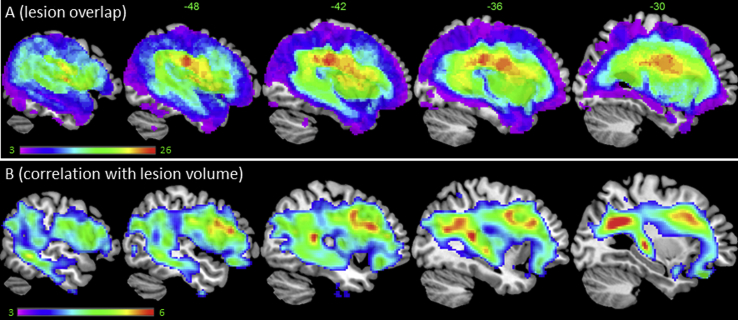
(A) Lesion overlap map across the 31 patients (threshold 1–26). (B) VBCM between regional integrity and lesion volume (*t*-scale 3–6).

Structural MRI scans were pre-processed with Statistical Parametric Mapping software (SPM8: Wellcome Trust Centre for Neuroimaging, http://www.fil.ion.ucl.ac.uk/spm/). The images were normalised into standard Montreal Neurological Institute (MNI) space using a modified unified segmentation–normalisation procedure optimised for focal lesioned brains (Seghier et al., 2008). Data from all participants with stroke aphasia and all healthy controls were entered into the segmentation–normalisation. This procedure combines segmentation, bias correction and spatial normalisation through the inversion of a single unified model (see Ashburner & Friston, 2005 for more details). In brief, the unified model combines tissue class (with an additional tissue class for abnormal voxels), intensity bias and non-linear warping into the same probabilistic models that are assumed to generate subject-specific images. Images were then smoothed with an 8 mm full-width-half-maximum (FWHM) Gaussian kernel and used in the lesion analyses described below. The lesion of each patient was automatically identified using an outlier detection algorithm, compared to healthy controls, based on fuzzy clustering. The default parameters were used apart from the lesion definition ‘U-threshold’, which was set to .5 to create a binary lesion image. We modified the U-threshold from .3 to .5 after comparing the results obtained from a sample of patients to what would be nominated as lesioned tissue by an expert neurologist. The images generated for each patient were individually checked and visually inspected with respect to the original scan, and were used to create the lesion overlap map in Fig. 1 and the individual lesion outlines in Fig. 3. We selected the Seghier et al. (2008) method as it is objective and efficient for a large sample of patients (Wilke, de Haan, Juenger, & Karnath, 2011), in comparison to a labour intensive hand-traced lesion mask. The method has been shown to have a Dice overlap >.64 with manual segmentation of the lesion and >.7 with a simulated ‘real’ lesion (where real lesions are superimposed onto healthy brains; Seghier et al., 2008). We should note here, explicitly, that although commonly referred to as an automated ‘lesion’ segmentation method, the technique detects areas of unexpected tissue class – and, thus, identifies missing grey and white matter but also areas of augmented cerebral spinal fluid (CSF) space.

We used the T1-weighted images with continuous signal intensity values across the whole brain and correlated these with individual measures or PCA factor scores using a voxel-based correlational methodology (VBCM: Tyler, Marslen-Wilson, & Stamatakis, 2005), a variant of voxel-lesion symptom mapping (VSLM: Bates et al., 2003). VBCM does not require a binary classification of the intact/lesioned brain to be marked, as in the case of VSLM, as both the behaviour and tissue concentration measures are treated as continuous variables (conducted in SPM8). Three analyses were conducted and all reported anatomical regions were located on the left hemisphere. First, each fluency measure was analysed separately, in an attempt to confirm the basic results from Borovsky et al. (2007). Secondly, the factor scores from the first speech production PCA were simultaneously entered to investigate how variation in tissue concentration corresponded to the unique components of speech dys-/fluency. Finally, the participants' factor scores from the omnibus PCA of the entire neuropsychological test battery and speech measures were entered into a VBCM analysis. In order to ensure that the results were not merely attributable to lesion size, each participants' lesion volume was calculated from the lesion identified by the automated lesion identification method (Seghier et al., 2008) and this was entered as a covariate in each VBCM. Hence, all analyses were performed with and without a correction for lesion volume. All anatomical labels were based on the Harvard–Oxford atlas in MNI space.

## 3 Results

### 3.1 Neuropsychological and lesion profiles

Table 2 summarises the participants' scores on the speech production measures and a large neuropsychological battery (Butler et al., 2014) and is ordered according to patients' scores on the BNT. A lesion overlap map for stroke aphasic participants is provided in Fig. 1A, and primarily covers the left hemisphere area supplied by the MCA (Phan et al., 2005). The maximum number of participants who had a lesion in any one voxel was 26 (−45, −23, 27; ventral portion of the postcentral gyrus).

### 3.2 Confirmation of previous basic findings on speech fluency alone

A bivariate correlation analysis on the speech production measures showed a high degree of inter-correlation. The token measure was correlated with MLU and WPM (*r* = .633, *p* < .001 and *r* = .513, *p* < .001 respectively). The MLU measure was correlated with TTR and WPM (*r* = .505, *p* = .004 and *r* = .674, *p* < .001 respectively). No other correlations reached significance. As noted by Borovsky et al. (2007), it is not unexpected to find inter-correlations between these measures. Indeed, the shared variance could reflect common factors underlying speech, including semantics, phonology and general aphasia severity. As highlighted in the Introduction, these high inter-correlations provide a challenge when attempting to relate the behavioural measures to unique aspects of the patients' lesions (see below).

**Fig. 2:**
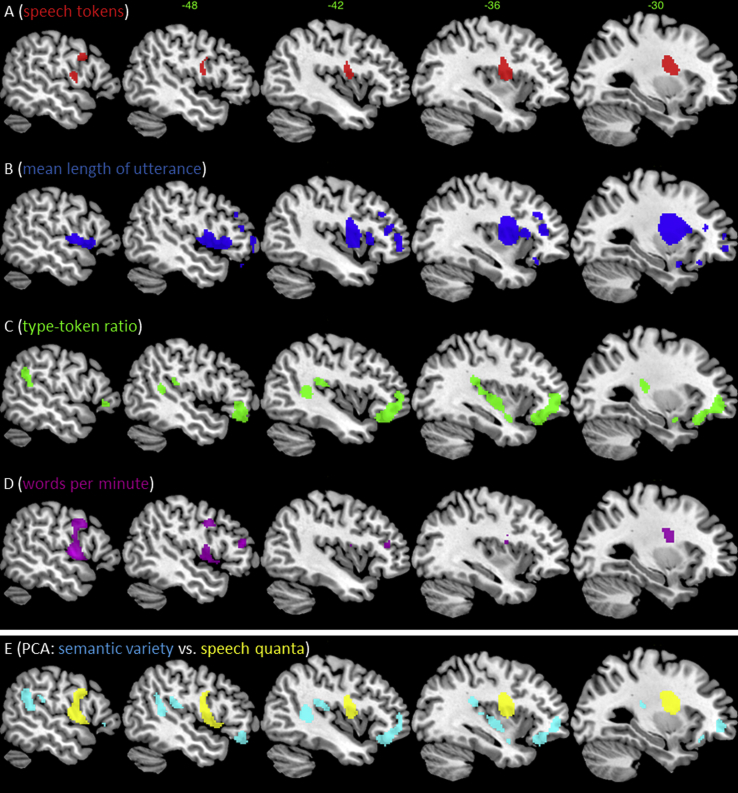
VBCM analysis using each speech production measure as the dependent measure [each *t*-map (scale 3) is thresholded at *p* < .005, cluster corrected at FWE of *p* < .001; without lesion volume correction]. Note each result reflects the raw (non-unique) correlation with each speech fluency measure (see text). (A) Lesion correlates for T. (B) Lesion correlates for mean length per utterance, (C) TTR. (D) Lesion correlates for WPM (uncorrected threshold *p* = .01, voxel-extent 50). (E) Lesion correlates of the unique PCA factors: F1 reflects speech quanta (yellow) and F2 reflects semantic variety (cyan). Note – no significant cluster remains for any factor after lesion volume correction (see text for further details & Fig. 1B).

The first VBCM analysis examined the neural correlates of fluency (T), complexity (MLU), semantic variety (TTR) and WPM each entered separately (Fig. 2A–D). The results for T revealed involvement of the superior-posterior insula and portions of the frontal lobe including the pre-central gyrus and middle frontal gyrus. The status of underlying white matter was also correlated. The analysis for MLU revealed an overlapping albeit broader region, extending throughout the left inferior frontal gyrus (IFG), insula and putamen, along the SMA, motor cortex and the underlying white matter.

The results for TTR, contrasted with those for T and MLU, in revealing a region that extended from supermarginal gyrus (SMG) and pMTG/STG through to aSTG as well as a portion of the anterior/orbito frontal gyrus. Peak voxels were in the IFG/frontal pole, SMG and posterior superior temporal gyrus (pSTG). Finally, the analysis for WPM revealed no significant correlations with lesion areas, although at an uncorrected voxel height of *p* = .01 (cluster extent 50 voxels) the results were very similar to those obtained for T.

### 3.3 Effect of lesion size

Given that some regions are more likely than others to be damaged after MCA stroke (Phan et al., 2005), we explored the intrinsic relationship between lesion location and size, and then controlled for this factor in the subsequent lesion–symptom analyses. Each participant's volume was calculated from the lesion identified by the modified segmentation–normalisation procedure. Lesion volume itself was correlated with the integrity of various regions with the MCA territory (Fig. 1B), representing the outer belt of the lesion overlap map (Fig. 1A). Crucially, including lesion size in the VBCM analysis for each speech measure reduced the significance across all measures and the underlying neural correlates (see Fig. 2A–D). The TTR and T measures did not correlate with any regions after accounting for lesion volume (even at a reduced image threshold of *p* < .01, FWE-cluster corrected *p* < .05). MLU was the only measure to reveal significant relationships with the left superior insula, putamen and underlying white matter after accounting for lesion volume.

### 3.4 Identifying principal speech-production components

The speech production measures were subjected to a rotated PCA and produced a two independent factor solution, which accounted for 84.65% of variance in participants' performance (F1 = 59.28%; F2 = 25.35%). The factor loading of each production measure is given in Table 3. The measures that tapped into overall quantity of speech (e.g., number of words and WPM) loaded heavily on factor 1; hence we refer to this factor as ‘speech quanta’. Factor 2 was interpreted as ‘semantic variety’, as the TTR measurement loaded heavily on it (e.g., proportion of unique words spoken). The MLU loaded heavily on factor 1, while loading to a lesser degree on factor 2. Lesion analysis for each factor, entered simultaneously, is displayed in Fig. 2E. The speech quanta measure (F1) was uniquely correlated with the left pre-central gyrus, superior insula and putamen, and the underlying white matter (including the superior longitudinal fasciculus, caudate–premotor tracts and the frontal ‘aslant’ tract (FAT) that connects the medial-superior portion of the frontal lobe to the inferior-lateral frontal region) (Basilakos et al., 2014, Catani et al., 2012, Rojkova et al., 2015). The semantic variety factor (F2) was uniquely correlated with the SMG and pMTG/STG plus the anterior/orbito frontal gyrus. Weaker correlation is observed along the STG to anterior lateral portions. After adding a covariate for lesion volume, no significant clusters remained for F1 or F2.

### 3.5 Identifying principal speech, language and executive behavioural factors

In order to determine the relationship between speech production measures and a range of language and cognitive skills, the factor scores from the speech PCA were correlated with the factor scores for phonology, semantics and executive abilities, computed in our previous study (Butler et al., 2014). The speech quanta factor only correlated with the first (phonology) factor (*r* = .404, *p* = .01). The semantic variety speech factor only correlated with the second (semantics) factor (*r* = .452, *p* < .001). To understand the relationship between speech fluency and various language and cognitive measures more thoroughly, an omnibus PCA was conducted. This resulted in a four factor solution including phonological skills (54.8% variance), semantic ability (11.6% variance) and cognitive–executive function (8.3% variance). We note here that a variety of different tasks over-and-above the executive tests loaded on this third factor. These include measures associated with auditory–phonological processing (e.g., minimal pairs) or semantic memory (e.g., CCT), although in both cases there was additional high loadings on the component one would typically associate with these tests. This outcome probably reflects the fact that the PCA process ‘decomposes’ the components of each task (reflecting the fact that no task is a pure measure of a single underpinning cognitive function). In this case, all the tasks that load onto the third factor require a high degree of additional ‘problem-solving’ and executively-loaded working memory.

**Fig. 3:**
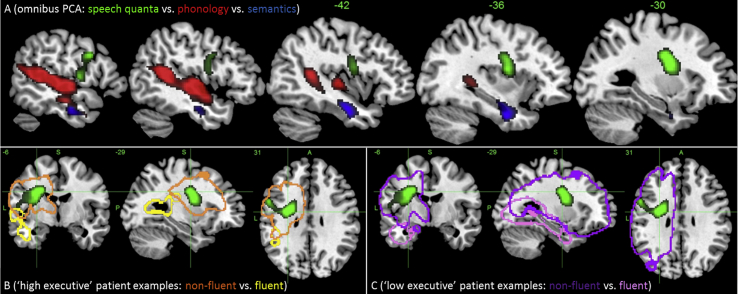
(A) VBCM analysis showing significant and unique neural correlates to phonological (red), semantic (blue) and speech quanta (green) performance, including lesion volume covariate. Image thresholded at *p* < .005, cluster corrected at FWE of *p* < .01 [image scale (*t*) 3–5]. Illustrative exemplar cases are shown in panels B and C. Panel B (patients with good executive scores) – patient EB (yellow) with good fluency versus KW (orange) with poor fluency. Panel C (patients with poor executive skills) – patient KS (pink) with good fluency versus ESb (purple) with poor fluency.

In this omnibus PCA, the ‘semantic variety’ characteristics of speech production were subsumed into the general semantics factor and a new additional fourth factor emerged based entirely on ‘speech quanta’ (6.3% variance) (see Table 4 for factor loadings and Table 5 for the patients' factors scores). The VBCM analysis for each independent factor, entered simultaneously, is shown in Fig. 3A and peak MNI co-ordinates are shown in Table 6. Each cluster shows where tissue concentration covaries uniquely with a given factor score, after accounting for lesion volume (the results without the lesion volume covariate were very similar). Results are thresholded at *p* < .005 voxel-level, *p* ≤ .001 family-wise error (FWE)-corrected cluster-level.

Performance on the phonological factor was uniquely correlated with voxels across a number of left hemisphere regions: primary auditory cortex (Brodmann area 41 and 42); mid to posterior middle and superior temporal gyri (MTG and STG); superior temporal sulcus (STS); and posterior portions of the insula, Heschl's gyrus and the planum temporale. The phonological cluster also overlapped with white matter regions, encompassing part of the arcuate fasciculus, a key aspect of the dorsal language pathway (Catani and ffytche, 2005, Catani et al., 2005, Duffau et al., 2005, Parker et al., 2005, Saur et al., 2008).

Performance on the semantic factor was uniquely related to a cluster in the left hemisphere anterior temporal lobe (ATL; see Fig. 3A). The cluster overlapped with the anterior-MTG and the temporal stem (including the dorsal edges of the inferior temporal gyrus – ITG and FG). Thus, with regards to white matter, the cluster included part of the ventral language route, overlapping with the inferior longitudinal fasciculus, inferior fronto-occipital fasciculus and uncinate fasciculus (Duffau et al., 2009, Mummery et al., 1999, Parker et al., 2005, Saur et al., 2008).

Performance on the speech quanta factor was uniquely related to a cluster in the left hemisphere pre-central gyrus, superior insula and putamen, extending medially to the caudate nucleus. With regards to white matter, the cluster included an area corresponding to the anterior part of the dorsal language route (superior longitudinal fasciculus) as well as the caudate–premotor tracts and the FAT (Rojkova et al., 2015). It is notable that the effect of lesion size on the speech quanta factor when calculated using all neuropsychological scores is negligible which was not the case when using the speech production measures alone (see above, Figs. 2E and 3A).

In contrast to the phonological, semantic and fluency factors, there were no clusters that correlated uniquely with the executive factor score which survived correction for multiple comparisons. This factor did, however, correlate with lesion volume in a simple correlation [*r*(31) = −.373, *p* = .039], suggesting that larger lesions result in poorer executive ability.

### 3.6 Individual cases

The relationship of individual patients to the group-level analyses was explored for two reasons. First, to test clinical utility, it is important to explore how individual results relate to the group-level maps for each language–cognitive factor. Secondly, exemplar cases help interpretation of the behavioural factors and their neural correlates (given that PCA, by design, generates scores that are one step removed from raw clinical measures: see Lambon Ralph et al., 2003). To achieve this aim, the group was divided into two using a median split based on executive factor scores to cover a range of patient types. Two participants were selected from each split to provide contrasting pairs who scored high or low on the speech quanta factor (see Fig. 3B and C). In the high executive group, patient KW (Broca's aphasia) had the lowest speech quanta score (orange outline) and EB (anomic) had the highest (yellow outline). In the low executive group, patient ESb (global) had the lowest speech quanta score (purple outline) and KS (transcortical sensory aphasia) the highest (pink outline). It is clear and encouraging from these examples that the individual patient lesions and associated behavioural profiles both align with the group-level VBCM results and are stable regardless of overall executive functioning (as should be expected given that the PCA approach extracts statistically-independent factors).

## 4 Discussion

Mirroring the principal features of natural language, speech fluency, phonological and semantic deficits are common following left MCA stroke. In most aphasia classification systems, patients are first divided by speech fluency followed by the relative balance of the patients' phonological and semantic abilities (Goodglass and Kaplan, 1983, Kertesz, 1982). By measuring and applying novel analyses to the overall rate, complexity and variety of naturalistic speech simultaneously with other measures of language performance, we were able to provide insights into their neural substrates. The results provide novel insights not only about the core features and neural bases of naturalistic language, but also how these underpin the varieties and nature of post-stroke aphasia.

In the first analysis, we largely confirmed the basic speech fluency results from Borovsky et al. (2007), by showing that the rate of T and MLU in the patients' speech correlated with the insula, SMA and underlying white matter. In contrast, TTR correlated with the SMG, pMTG, aSTG, and anterior/orbito frontal gyrus. Whilst these results are interesting and support previous observations that speech fluency is a multi-faceted aphasic feature (Ackermann and Riecker, 2004, Basilakos et al., 2014), their interpretation is complicated by the fact that these behaviour–lesion correlations were not significant when lesion volume was controlled and no account is taken of the patients' other aphasic features – thus the picture of the lesion–symptom mappings is incomplete.

We addressed these limitations by utilising the PCA-VBCM methodology in the two subsequent stages. We initially undertook a PCA of the speech production measures alone. This PCA suggested that the clinical concept of ‘fluency’ reflects two more general and independent factors: ‘speech quanta’ (the quantity of speech produced) and ‘variety’ (the range of different words/token irrespective of the quantity produced). Scores from the speech quanta factor correlated uniquely with tissue damage in the left pre-central gyrus, superior insula and putamen, and the neighbouring white matter (including the superior longitudinal fasciculus, caudate–premotor tracts and the FAT that connects the medial-superior portion of the frontal lobe to the inferior-lateral frontal region) (Rojkova et al., 2015). In contrast, the speech variety factor was uniquely related to the SMG and pMTG/STG plus the anterior/orbito frontal gyrus. However, again the results for these speech production-only data were largely confounded with lesion volume.

These twin challenges of inter-correlation and severity were overcome in the final step in which we completed an omnibus PCA containing all the detailed behavioural data and the speech production measures, which also allowed us to explore the relationship between speech fluency and other aphasic features (Basilakos et al., 2014). This analysis revealed four unique factors (phonology, executive processing, semantic function which included the speech variety measure, and speech quanta). The phonological factor uniquely correlated with damage to left perisylvian areas, including left mid to posterior STG, MTG, STS and Heschl's gyrus (HG), as well as the underlying white matter (arcuate fasciculus portion of the dorsal language pathway). The semantic factor correlated uniquely with left anterior-MTG and the underlying temporal stem. The speech quanta factor correlated uniquely with left hemisphere pre-central gyrus, superior insula and putamen, extending medially to the caudate nucleus. With regards to white matter, the cluster included an area corresponding to the anterior part of the dorsal language route (superior longitudinal fasciculus) as well as the caudate–premotor tracts and the FAT. The executive factor did not covary, uniquely, with any brain regions, as found previously (Butler et al., 2014).

**Fig. 4:**
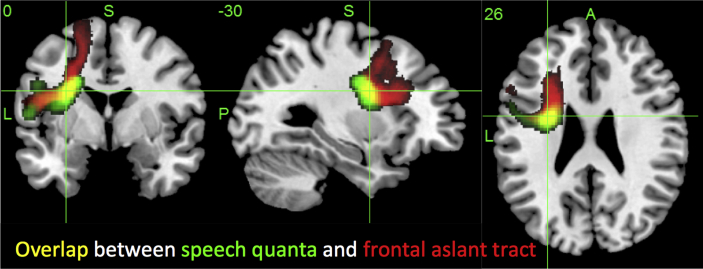
Overlap (yellow) between the speech quanta-related lesion correlate (green) and the FAT (red). The speech quanta image is thresholded at *p* < .005, FWE of *p* < .01 [image scale (*t*) 3–5], and the FAT is a probabilistic map (image scale .5–1).

The speech quanta-related region included key cortical and subcortical areas associated with deficits of articulatory planning (Bates et al., 2003, Borovsky et al., 2007, Dronkers, 1996) and motor coordination of speech related movements (Ackermann & Riecker, 2004). Likewise, direct stimulation of ventral PMC and pars opercularis leads to speech inhibition (Kinoshita et al., 2014). The underlying white matter damage might also be crucial and, indeed, the core of the identified speech quanta-related region overlaps with the FAT (Rojkova et al., 2015; see Fig. 4). The integrity of this tract has been shown to correlate with non-fluent speech in primary progressive aphasia (Catani et al., 2013, Mandelli et al., 2014) and, when combined with the integrity of the anterior section of the arcuate fasciculus, to correlate with clinician-rated fluency in post-stroke aphasia (Basilakos et al., 2014). In keeping with our finding of a separate principal component for speech quanta over and above phonology and semantic skills, Catani et al. (2013) did not find a relationship between FAT integrity and repetition or syntactic abilities in primary progressive aphasia. Additional convergent evidence has been derived from neurosurgical investigations: direct electrical stimulation of FAT inhibits speech and subsequent surgical resection leads to transient postoperative inhibition (Kinoshita et al., 2014).

The results for the phonological, semantic and executive factors reproduced those observed in our previous study and related investigations (Butler et al., 2014, Mirman et al., 2015a, Mirman et al., 2015b). It is interesting to note that speech ‘variety’ seems to be inherently linked to the patients' degree of remaining semantic abilities (factor 2) presumably because the use of a wide vocabulary is underpinned by fine semantic distinctions. More generally, the observed neural correlates of the phonological and semantic factors are highly consistent with data from other methods. Phonological processing was associated with left HG, mid to posterior MTG, STG and STS, which have all been identified in various previous reviews of phonological processing (Hickok and Poeppel, 2004, Hickok and Poeppel, 2007, Price, 2010, Price, 2012, Vigneau et al., 2006, Wise et al., 2001) and repetitive transcranial magnetic stimulation (rTMS) to pSTG has been shown to increase error rates in the speech production of neurologically-intact participants (Acheson, Hamidi, Binder, & Postle, 2011). The neural substrate for semantic performance (left anterior temporal regions) again converges with findings from large-scale functional magnetic resonance imaging (fMRI) reviews (Binder et al., 2009, Price, 2010, Vigneau et al., 2006, Visser et al., 2009), data from semantic dementia (Mion et al., 2010), rTMS (Pobric, Jefferies, & Lambon Ralph, 2007), direct electrical stimulation of inferior fronto-occipital fasciculus (IFOF) (Duffau, Gatignol, Mandonnet, Capelle, & Taillandier, 2008) and neuroanatomically-constrained models (Ueno, Saito, Rogers, & Lambon Ralph, 2011).

One thing that must be kept in mind when using PCA is that the factor solution is always dependent on the tests included in the decomposition. Thus, if an analysis fails to include behavioural measures that reflect a core feature of the target clinical populations then, by definition, the resultant PCA model will not derive a principal dimension for this clinically-relevant feature. Our current PCA model contains measures of phonological, semantic, speech fluency and executive skill. Whilst this model captures some of the core aspects of post-stroke aphasia, future studies will need to augment the range of assessments in order to capture other key features of this clinical disorder including syntactic processing, speech errors, written language processing, etc.

To conclude, in addition to revealing specific behavioural and neuroanatomical information about the nature of chronic post-stroke aphasia, the current study serves as a worked example for the utility of our PCA-VBCM method. Rather than adopting either a classical group-study or single-case investigation, the PCA data-driven approach not only generates a single unified model for the group as a whole (expressed in terms of the four emergent principal components) but is also able to capture the individual differences between patients (in terms of their relative positions along the principal behavioural axes). This method not only preserves the individual-level data (as per single-case and case-series methods) but is also able to place it within the broader context of the whole group (akin to group studies). Thus rather than ignoring or averaging across individual variations, the methodology actively embraces individual differences and extracts coherent variations. We also note here that by utilising varimax rotation, the emergent factors become relatively straightforward to interpret from a cognitive perspective and the fact that they are statistically-independent makes them ideal for the neuroimaging analyses (which call for orthogonal predictors rather than the strong inter-correlations that are found between the raw test results). Whilst we have applied this PCA-VBCM approach to post-stroke chronic aphasia in the current investigation, this methodological approach should be applicable and beneficial across a range of acute and progressive neurological conditions.

## Figures and Tables

**Table 1:** Participant background information. Cases are ordered according to BNT score.

	Initials	Age (years)	Gender	Years of education	Time post-stroke (months)	Lesion volume (voxels)	BDAE classification
1	DBb	66	M	12	59	42,687	Wernicke
2	ES	69	M	11	39	28,146	Global
3	ESb	68	M	11	142	33,193	Global
4	KW	81	M	10	24	11,393	Broca
5	BS	59	M	11	103	27,242	Broca
6	KL	55	M	13	31	14,625	Mixed non-fluent
7	LM	63	M	11	13	14,990	Global
8	DB	60	M	12	44	31,644	Wernicke
9	PE	73	F	16	22	6959	Wernicke/conduction
10	KS	59	M	12	12	5822	Transcortical sensory aphasia
11	KK	48	M	12	33	20,043	Broca
12	WM	77	M	11	66	33,282	Mixed non-fluent
13	GL	47	M	12	18	26,319	Broca
14	DCS	45	F	12	12	5273	Broca
15	JSa	73	M	11	190	45,875	Mixed non-fluent
16	JSc	78	M	12	76	18,459	Broca
17	JA	65	M	11	128	26,097	Mixed non-fluent
18	JJ	84	M	12	25	8951	Anomia
19	JM	62	M	11	110	15,492	Anomia
20	JSb	72	M	11	23	1481	Anomia
21	ER	64	F	14	181	26,480	Mixed non-fluent
22	HN	81	M	10	56	25,963	Anomia
23	BH	64	M	11	26	8795	Mixed non-fluent
24	EB	61	M	17	12	4806	Anomia
25	DM	49	M	17	42	11,915	Broca
26	DS	72	M	11	106	11,446	Transcortical motor aphasia
27	AG	55	M	11	131	21,270	Broca
28	LH	65	M	11	81	10,073	Anomia
29	JMf	70	F	11	84	8921	Anomia
30	AL	49	F	12	69	9767	Anomia
31	TJ	60	M	12	23	19,975	Anomia

**Table 2:** Participant scores on the behavioural assessment battery and speech production measures.

ID	NW rep I	NW rep d	W rep I	W rep d	Picture naming	Boston naming	NW min pairs	W min pairs	SWPM	WWPM	CAT–sentence	Synonym	CCT	Brixton	Ravens	Digit F	Digit B	WPM	TTR	MLU	T
DBb	0	0	37.5	0	0	0	22.22	52.78	57.81	31.25	12.5	48.96	53.13	38.18	30.56	25	0	63.33	53.91	60.74	23.89
ES	0	0	0	0	4.69	0	48.61	54.17	78.13	90.63	25	72.92	73.44	40	66.67	0	0	15.12	94.09	35.19	24.63
ESb	0	0	0	0	0	0	54.17	50	87.5	60.94	34.38	52.08	43.75	23.64	38.89	0	0	0	0	0	0
KW	0	0	3.75	0	1.56	0	75	65.28	95.31	92.19	84.38	82.29	89.06	50.91	80.56	50	42.86	2	86.25	12.35	2.99
BS	3.33	0	5	1.25	3.13	1.67	65.28	75	92.19	100	31.25	78.13	84.38	38.18	91.67	0	0	52.61	63.45	48.15	21.65
KL	0	0	6.25	0	4.69	1.67	75	77.78	92.19	98.44	28.13	68.75	78.13	61.82	88.89	0	0	11.87	44.72	21.16	13.44
LM	13.33	3.33	27.5	0	1.56	1.67	43.06	54.17	67.19	53.13	28.13	57.29	68.75	32.73	61.11	0	0	14.51	46	18.52	7.47
DB	70	30	85	83.75	7.81	8.33	87.5	58.33	64.06	76.56	31.25	59.38	82.81	40	86.11	37.5	14.29	17.42	38.33	65.36	89.58
PE	13.33	3.33	45	41.25	20.31	11.67	77.78	86.11	96.88	100	50	79.17	84.38	41.82	80.56	25	28.57	49.1	60.62	93.7	100
KS	73.33	80	93.75	95	31.25	13.33	94.44	95.83	71.88	67.19	84.38	84.38	68.75	52.73	86.11	100	57.14	70.53	86.6	90.53	60.47
KK	33.33	3.33	56.25	26.25	42.19	15	72.22	95.83	93.75	95.31	46.88	81.25	84.38	76.36	100	0	0	14.75	62.21	50	45.54
WM	36.67	30	55	41.25	39.06	25	47.22	63.89	92.19	75	50	61.46	51.56	43.64	61.11	37.5	28.57	5.44	65.71	22.22	10.45
GL	93.33	63.33	100	81.25	68.75	31.67	98.61	97.22	96.88	95.31	65.63	75	73.44	58.18	91.67	37.5	28.57	9.24	55.86	29.97	52.26
DCS	40	56.67	72.5	68.75	67.19	43.33	97.22	97.22	100	98.44	93.75	91.67	95.31	81.82	100	62.5	57.14	14.83	92.74	70.37	23.14
JSa	30	3.33	75	65	62.5	46.67	75	77.78	92.19	98.44	59.38	81.25	76.56	67.27	83.33	50	28.57	30.84	74.41	49.38	25.38
JSc	36.67	63.33	90	91.25	71.88	53.33	75	86.11	98.44	98.44	75	76.04	82.81	43.64	77.78	62.5	42.86	23.9	90.36	61.11	20.9
JA	36.67	40	85	78.75	79.69	63.33	90.28	95.83	100	98.44	78.13	63.54	87.5	61.82	80.56	37.5	0	46.26	74.41	60.74	25.38
JJ	36.67	23.33	82.5	73.75	85.94	63.33	51.39	80.56	98.44	98.44	56.25	93.75	53.13	43.64	41.67	62.5	42.86	41.98	85.19	82.72	40.31
JM	83.33	83.33	100	98.75	81.25	63.33	93.06	95.83	100	100	100	82.29	81.25	76.36	94.44	50	57.14	79.16	77.63	84.77	59.72
JSb	63.33	36.67	86.25	81.25	75	63.33	76.39	88.89	93.75	90.63	84.38	75	78.13	60	86.11	62.5	28.57	41.18	92	47.62	26.13
ER	53.33	36.67	70	81.25	71.88	65	81.94	88.89	95.31	93.75	56.25	84.38	90.63	41.82	38.89	25	0	46.49	82.14	95.06	47.03
HN	36.67	23.33	83.75	80	65.63	65	77.78	76.39	93.75	93.75	37.5	85.42	85.94	25.45	75	50	42.86	25.4	91.27	91.36	47.03
BH	86.67	80	100	96.25	95.31	66.67	93.06	94.44	98.44	93.75	78.13	83.33	73.44	67.27	66.67	62.5	57.14	36.77	78.68	60.49	28.37
EB	83.33	53.33	100	100	81.25	66.67	94.44	98.61	98.44	100	71.88	94.79	90.63	80	100	75	57.14	78.87	67.16	98.52	93.31
DM	60	10	73.75	68.75	75	71.67	80.56	93.06	98.44	98.44	56.25	95.83	98.44	50.91	91.67	37.5	0	23.63	84.74	50.79	28.37
DS	56.67	33.33	88.75	91.25	84.38	73.33	79.17	77.78	100	100	87.5	93.75	89.06	72.73	72.22	50	28.57	34.53	100	100	17.17
AG	73.33	83.33	77.5	87.5	87.5	78.33	100	98.61	100	100	87.5	89.58	75	56.36	75	100	100	13.06	80.5	54.81	22.4
LH	56.67	50	82.5	88.75	81.25	78.33	95.83	97.22	96.88	100	90.63	92.71	87.5	76.36	88.89	87.5	57.14	66.11	74.33	52.91	61.21
JMf	93.33	66.67	96.25	98.75	96.88	80	90.28	95.83	100	100	71.88	91.67	93.75	50.91	83.33	62.5	57.14	50.53	69	73.15	48.52
AL	90	90	100	98.75	93.75	88.33	91.67	100	100	100	84.38	93.75	79.69	60	91.67	87.5	85.71	100	86.25	87.65	44.79
TJ	93.33	83.33	98.75	92.5	95.31	95	87.5	98.61	98.44	100	68.75	88.54	70.31	52.73	50	75	28.57	38.28	76.67	81.48	38.07

Abbreviations: W – word; NW – non-word; rep I – immediate repetition; rep D – delayed repetition; S/WWPM – spoken/written word–picture matching; Digit F – forward digit span; Digit B – backward digit span.

CAT column refers to spoken sentence comprehension subtest.

**Table 3:** Factor loadings for PCA of speech production measures.

Measure	F1	F2
TTR	.082	**.973**
MLU	**.803**	.488
T	**.904**	−.157
WPM	**.800**	.258

Footnote: Factor loadings exceeding .5 are marked in bold.

**Table 4:** Factor loadings from the omnibus PCA.

	1	2	3	4
Non-word repetition immediate	**.822**	.108	.220	.326
Non-word repetition delayed	**.912**	.106	.126	.175
Word repetition immediate	**.780**	.230	.122	.430
Word repetition delayed	**.783**	.315	.168	.411
Forward digit span	**.841**	.239	.058	.198
Backward digit span	**.774**	.191	.166	.070
Non-word minimal pairs	**.584**	.203	**.683**	.167
Word minimal pairs	**.586**	.421	**.505**	.237
CAT – sentence	**.726**	.388	.374	−.050
Cambridge 64-item naming	**.712**	**.600**	.096	.168
BNT	**.659**	**.629**	.013	.209

96-Synonym judgement	.353	**.714**	.347	.244
Spoken word-to-picture matching	.311	**.722**	.378	−.190
Written word-to-picture matching	.156	**.699**	**.578**	.022

Camel and Cactus – pictures	−.078	.461	**.702**	.276
Raven's Coloured Progressive	.091	.034	**.918**	.079
Brixton spatial anticipation test	.391	.232	**.629**	.018

TTR	.235	**.749**	.055	.160
WPM	.310	.103	.013	**.716**
Mean length per utterance	.294	.397	.000	**.814**
T	.154	−.152	.350	**.818**

Footnote: Factor loadings exceeding .5 are marked in bold.

**Table 5:** PCA factor scores from Butler et al. (2014), fluency-only PCA and the omnibus PCA.

	Butler et al. (2014)			Speech PCA		All scores combined			
	F1-phon	F2-sem	F3-exe	F1-flu	F2-var	F1	F2	F3	F4
DBb	−.332	−2.321	−2.018	.290	−.499	−.775	−1.600	−2.606	1.043
ES	−1.668	−.266	−.331	−1.024	.797	−1.842	.384	−.564	−.256
ESb	−1.078	−.701	−1.911	−1.360	−3.030	−.300	−1.773	−1.290	−1.923
KW	−1.211	−.202	.972	−1.853	.540	−.832	.387	.700	−1.929
BS	−1.935	.267	.678	−.136	−.217	−1.952	.249	.604	.120
KL	−1.810	−.072	.972	−1.046	−1.185	−1.391	−.516	1.193	−1.175
LM	−.873	−1.682	−.765	−1.171	−1.072	−.843	−1.445	−.946	−.606
DB	.315	−2.295	.594	1.151	−2.058	−.149	−2.555	.658	1.491
PE	−1.067	.358	.459	1.961	−.980	−1.426	−.090	.706	1.723
KS	1.726	−2.395	.718	1.209	.616	1.274	−1.524	−.015	1.274
KK	−1.207	.161	1.319	−.164	−.642	−1.129	−.158	1.504	−.149
WM	−.036	−.525	−1.344	−1.364	−.297	.363	−.567	−1.198	−1.458
GL	.618	−.361	.583	−.284	−1.181	.971	−1.148	1.107	−.715
DCS	.208	.045	1.680	−.642	1.080	.346	.564	1.351	−.819
JSa	−.321	.309	.233	−.453	.148	−.240	.358	.221	−.398
JSc	.416	.356	−.256	−.643	.947	.461	.657	−.344	−.500
JA	−.124	.593	.308	−.099	.279	.009	.377	.417	−.170
JJ	.236	1.429	−2.197	.307	.706	.131	1.420	−2.027	.348
JM	.993	−.053	.863	1.328	.227	.998	−.210	.805	.683
JSb	.472	.006	.120	−.456	.854	.537	.269	.003	−.409
ER	−.241	1.337	−1.092	.677	.612	−.622	1.273	−.957	1.189
HN	−.089	.712	−.692	.253	.909	−.534	1.048	−.754	.927
BH	1.204	.216	−.296	−.214	.392	1.422	.090	−.198	−.522
EB	.760	.174	1.150	2.256	−.526	.451	−.204	1.215	1.739
DM	−.607	1.269	.420	−.566	.525	−.727	1.239	.551	−.013
DS	.128	1.045	.041	−.199	1.760	−.051	1.653	−.282	.129
AG	1.419	.210	−.064	−.759	.451	1.810	.244	−.018	−1.321
LH	.704	.354	.807	.830	−.240	.765	.101	.885	.234
JMf	.811	.737	.110	.624	−.131	.755	.391	.294	.467
AL	1.446	.262	.228	1.285	.842	1.353	.365	.000	.834
TJ	1.141	1.029	−1.289	.265	.373	1.165	.718	−1.013	.161

**Table 6:** Neural correlates for omnibus PCA factors after accounting for lesion volume.

Principal component	Location	Extent (voxels)	*Z*	MNI co-ordinates		
				*x*	*y*	*z*
Factor 1 (phonology)	Left temporal lobe	4387				
	Planum polare		4.24	−48	−16	0
	Posterior supramarginal gyrus		4.23	−48	−50	14
	Posterior middle temporal gyrus		4.06	−62	−18	−14

Factor 2 (semantic)	Left temporal lobe	1030				
	Anterior middle temporal gyrus		3.68	−60	−6	−26
	Anterior temporal fusiform cortex		3.63	−38	−6	−28
	Posterior ITG		2.77	−70	−24	−30

Factor 4 (speech quanta)	Left prefrontal lobe	2164				
	Corticospinal tract		3.85	−24	−4	32
	Pre-central gyrus		3.80	−30	−4	28
	Pre-central gyrus		3.58	−54	8	32
